# Physical Rehabilitation Services in Disasters and Emergencies: A Systematic Review

**Published:** 2019-05

**Authors:** Ghasem MOUSAVI, Ali ARDALAN, Hamidreza KHANKEH, Mohammad KAMALI, Abbas OSTADTAGHIZADEH

**Affiliations:** 1. Department of Health in Disasters and Emergencies, School of Public Health, Tehran University of Medical Sciences, Tehran, Iran; 2. Research Center for Disaster Recovery, University of Social Welfare & Rehabilitation Sciences, Tehran, Iran; 3. Department of Rehabilitation Basic Sciences, Rehabilitation Research Center, School of Rehabilitation Sciences, Iran University of Medical Sciences, Tehran, Iran

**Keywords:** Physical rehabilitation, Disability, Disasters, Emergencies

## Abstract

**Background:**

Physical rehabilitation, as one of the rehabilitation disciplines, can play a great role in humanitarian reliefs. The effectiveness of physical rehabilitation services is completely dependent on time of intervention, the importance of good timing in providing services during disasters is not well understood. The objective of this study was to systematically review the physical rehabilitation services provided in disasters and emergencies.

**Methods:**

An electronic search of PubMed, Scopus, Cochrane, and PEDro (Physiotherapy Evidence Database) was undertaken from Jan 2000 to Sep 2017. All English studies reporting physical rehabilitation services in natural and man-made disasters were selected regardless of study design. The included studies were analyzed by descriptive and analytical method.

**Results:**

Thirteen studies were included after reviewing by title, abstract and full text in this study. Most of the physical rehabilitation studies come back to recent years. Most of the disaster physical rehabilitation services were physiotherapy and occupational therapy. The physical rehabilitation experts have been attended in the affected area from the few first hours until several months after disasters in order to provide the required services to the affected population.

**Conclusion:**

There are few studies about physical rehabilitation services provided in the disaster-affected areas and this study showed that the services were limited and at different times. Physical rehabilitation services post disasters should have a comprehensive service model, like other health services. Therefore, it is necessary to conduct further studies to achieve this aim.

## Introduction

Many major health conditions need rehabilitation to improve outcomes following disasters and emergencies (1–3). Rehabilitation is “a set of measures that assist individuals who experience, or are likely to experience disability to achieve and maintain optimal functioning in interaction with their environments” (4). If rehabilitation intervention is provided in time, they can result in better health outcomes; reduce hospital stays and the probability of long term disability. Rehabilitation includes improving an individual’s ability to function and to impact his/her environment (4). Moreover, rehabilitation includes prevention of the loss of function, restoration of function and increase or maintenance of current function (4, 5). There is a lack of data on the global need for rehabilitation (4, 6). Rehabilitation has recently been recognized as an important sector of humanitarian response (7). Some of the injured people in disasters and emergencies experience short or long-term disability due to inadequate treatment of injuries (8). Bone fractures, spinal cord injuries, traumatic brain injuries, amputations, peripheral nerve injuries, and burns are common injuries in disasters and conflicts which can lead to physical or cognitive limitation in functioning in the victims (9–12).

Article 11 of the United Nations (UN) convention on the rights of persons with disabilities mandates countries to support persons with disability in disasters and conflicts (13). The UN convention mandates that rehabilitation interventions should occur during early disaster response and follow months and years later at the community level. Currently, rehabilitation services are rarely provided for victims of disasters, if they are, they tend to be not at the right time or place (14).

Physical rehabilitation plays an important role in the rehabilitation process and it is an essential part of fully integrating disabled people in society (15). Several studies have confirmed the effectiveness of early rehabilitation intervention for treating injured people in disasters (10, 16, 17). Moreover, persons with preexisting disabilities can benefit from rehabilitation services in affected areas (4, 14, 18). Many studies have reviewed rehabilitation services post-disasters such as Hurricane Katrina, the Pakistan flood, Sichuan and Haiti earthquakes and have found that unique rehabilitation intervention which relied completely upon researchers’ experiences and interests (16, 19–21). Literature on rehabilitation reliefs in disasters is scarce, anecdotal (22) and is dependent on the authors’ field experiences. Some rehabilitation experts have recommended conducting various scientific researches to gain a better understanding of the necessary rehabilitation reliefs provided in disasters (23).

Accordingly, this study aimed to investigate the physical rehabilitation services provided in disasters and emergencies.

## Methods

This study was conducted to answer this research question as follows: what are the characteristics (kind of service and time of service delivery) of physical rehabilitation services in provided in past disasters and emergencies?

### Data sources

For the purpose of this review, PubMed, Scopus, Cochrane, and PEDro (Physiotherapy Evidence Database) databases were the main sources of information in this study. Since research on disaster physical rehabilitation is a relatively new concept, the time of the search was limited to Jan 2000 to Sep 2017.

They were accessed and searched in Dec of 2017. In addition, for locating gray literature, we used different searching strategies: 1) Google search engine, 2) purposive websites, and 3) consultation with national and international experts. The reference lists of founded studies were other fruitful sources of material.

### Search strategy

All sources of written material were obtained using the same search strategy. Terms such as physical rehabilitation, physiotherapy, physical therapy, occupational therapy, speech therapy, orthotics, prosthetics, disaster, and conflict were applied by using Medical Subject Headings (MESH). MESH terms were also used to find more relevant articles (Table 1).

**Table 1:** Search strategy applied to search the databases

*A)(rehabilitation) OR (“physical rehabilitation”) OR (“physical therapy”) OR (physiotherapy) OR (“occupational therapy”) OR (“speech therapy”) OR (orthotics) OR (prosthetics)* *B)(disaster) OR (earthquake) OR (conflict) OR (emergency)* *C)(rehabilitation OR “physical rehabilitation” OR “physical therapy” OR physiotherapy OR “occupational therapy” OR “speech therapy” OR orthotics OR prosthetics) AND (disaster OR earthquake OR conflict OR emergency)*

### Inclusion criteria

Included articles were that published in academic journals, reported one or more kinds of physical rehabilitation intervention such as physiotherapy, occupational therapy, speech therapy, orthotics and prosthetics in natural/man-made disasters and conflicts.

### Exclusion criteria

Excluded articles focused only on the importance and effectiveness of rehabilitation services or the epidemiology of injuries of disaster and conflicts. Moreover, studies with no intervention about providing rehabilitation services in disasters and conflicts were removed from the study. Non-English studies and studies published before 2000 were also excluded.

### Study selection and data extraction

**Fig. 1:**
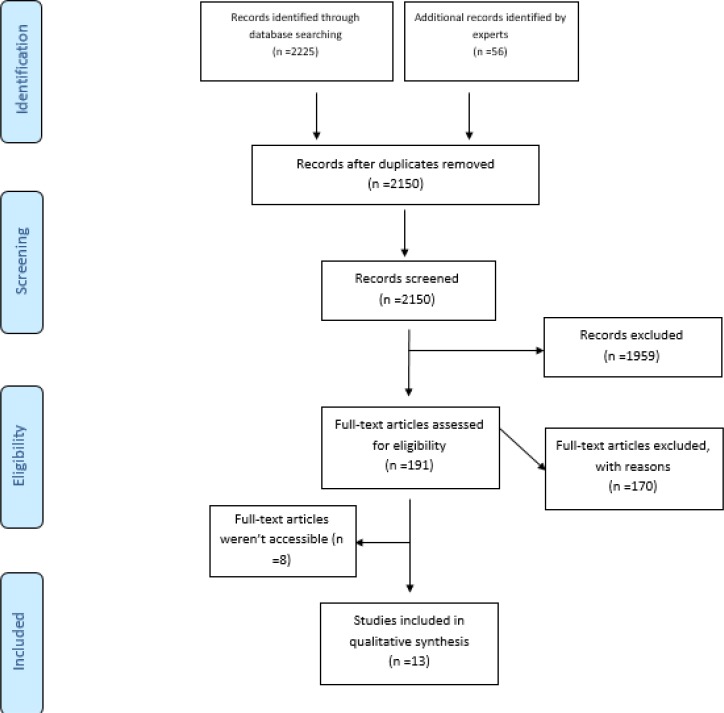
Flow diagram of the articles selecting process

The first author reviewed titles of all articles retrieved by searching in databases. Every article that met the inclusion criteria was selected. Selected documents were entered in Microsoft Excel Spreadsheet and duplicate titles were removed. Finally, all articles were reviewed by the second author. In order to perform a blind-review, the names of authors and journals were removed (Fig. 1). The reviewers applied a form to extract data from selected studies. The form included: name of the first/corresponding author, country, publication year and type of hazard, kind and time of intervention, methodology of the study and summary of findings. Then the information of included studies and training material were categorized to descriptive and thematic analysis.

## Results

Totally, 2281 studies were obtained through electronic search in databases and other resources. After removing duplicate articles, 2150 titles were selected for further review. By reviewing studies based on title and abstract, 1959 studies were excluded from the study and three abstracts were not found. Full-text review excluded 170 articles while eight full texts were inaccessible for review. Altogether, 13 qualified studies were analyzed in this review.

### Descriptive Analysis

Four articles of the thirteen (31%) were affiliated to China and Canada (2 articles per country), three articles were affiliated with USA (23%) and the 6 other countries, including Haiti, Netherlands, Iran, India, Australia and Philippines were attended with one article in this study (46%). Ten articles of the thirteen (76.9%) were published during 2010–2016. One study had cohort design, one study was with design of quasi-experimental and the two researches has been designed cross-sectional while the other nine resources were review of programs, experiences, clinical notes or perspective. Most studies (69.2%) focused on providing services of physical rehabilitation after the earthquake, two (15.4%) on hurricane and two (15.4%) were related to man-made disasters including bombing attack and conflict.

Overall, 11 of the 13 included studies (84.6%) were physical rehabilitation relevant services in past six major natural disasters, including Bam, Kashmir, Sichuan, Haiti and Nepal earthquakes and the Hurricane Katrina. Two (15.4%) studies focused on physical rehabilitation service delivery in man-made disasters. The earliest document in this review was about providing physical rehabilitation services after the Bali bombing disaster 2002 that published in 2005.

There was no publication on physical rehabilitation in disasters and emergencies in 2006. In 2007, two articles were published, Bam earthquake 2003, and Hurricane Katrina 2005, respectively. There was another gap between 2008 and 2009. However, in 2010 three articles were published in relation to the Haiti earthquake and establishing physical rehabilitation services to affected population. During 2012, 2013 and 2014, two, one and two articles were published, respectively. One article was published in 2015 and one article was published in 2016 (Table 2).

**Table 2:** Articles analyzed for the systematic review literature

***Authors***	***Year***	***Study type***	***Affiliated Country***	***National/International providers***	***Previously coordinated (Yes/NO)***	***Hazard***
Edgar D, Wood F, Goodwin-Walters A	2005	Report on experience	Australia	National	Y	Bombing
Raissi GR,	2007	Clinical note	Iran	National and international	N	Earthquake
Bloodworth D. M., Kevorkian C. G., Rumbaut E., Chiou-Tan F. Y.	2007	Review	USA	National	N	Hurricane
Iezzoni L.	2010	Perspective	USA	International	N	Earthquake
Landry M, O’Connell C, Tardif G, Burns A	2010	Perspective	Canada	International	N	Earthquake
Gorry C.	2010	International Cooperation Report	Haiti	International	Y	Earthquake
Li Y, Reinhardt J, Gosney J, Zhang X, Hu X, Chen S et al	2012	Prospective cohort study	China	National	N	Earthquake
O’Connell C, Ingersoll A	2012	Report on experience	Canada	National and international	N	Earthquake
Zhang X., Reinhardt J. D., Gosney J. E., Li J	2013	Longitudinal quasi-experimental	China	National	N	Earthquake
Armstrong JC, Nichols BE, Wilson JM, Cosico RA, Shanks L.	2014	Review of program outcomes	Netherlands	National and international	N	Civil conflict
Keshkar S, Kumar R, Bharti B	2014	Cross-Sectional, descriptive	India	National	N	Earthquake
Benigno MR, Kleinitz P, Calina L, Alcido MR, Gohy B, Hall JL	2015	Cross-Sectional, descriptive	Philippines	National and international	N	Typhoon
Landry MD, Sheppard PS, Leung K, Retis C, Salvador EC, Raman SR.	2016	perspective article	USA	National and international	N	Earthquake

### Analytical analysis

Most studies indicated the multidisciplinary physical rehabilitation including physical therapy, occupational therapy and assistive devices as the provided rehabilitation services in the earthquake zones. Two studies focused on physical therapy as an intervention, one study highlighted on prosthetics delivery in humanitarian action and the other research was related to orthotics and physiotherapy. In such disasters, the physical rehabilitation services were established temporary in periods of time and depended on existing cases in the affected area. In the other disasters, the provided rehabilitation services completely relied on modern and professional equipment that was incompatible with the conditions and facilities of the region before occurring disaster.

In addition, there is not a consensus on time of physical rehabilitation service delivery in disasters and emergencies. In some cases such as bombing attack, the mentioned services have been provided during hospitalization for victims, while in a conflict, access to rehabilitation services may postpone for several months. We observed in other studies that this time between the occurring of disaster and delivery of rehabilitation services varies from medical stabilization of the injured persons in first or two weeks to one or two months following disasters. In all studies, physical rehabilitation services were established by rehabilitation professionals. In the Bam earthquake, first, the international providers established rehabilitation services to affected local people and then the Iranian specialists were involved in the delivery of rehabilitation services. In relation to preparedness of physical rehabilitation relief teams, most of these teams that attended at the disaster scene were not previously prepared. Only, in one case the coordination between international organizations and national service providers were organized prior to disaster. Table 3 shows the rehabilitation services provided in past disasters and emergencies.

**Table 3:** Provided physical rehabilitation services during past disasters and emergencies

***Event***		***Kind of services***	***Time of services***
Earthquake	Bam (2003)	Physical medicine and rehabilitation, physical therapy, occupational therapy, orthotics and prosthetics	After at least one month
	Kashmir (2005)	Rehabilitation aids devices, physiotherapy and psychotherapy, Physical medicine and rehabilitation	Same time as the medical treatment services
	Sichuan (2008)	Physical medicine and rehabilitation, physiotherapy, occupational therapy and traditional Chinese medicine	Two months after the event
	Haiti (2010)	Rehabilitation services	First two weeks
	Nepal (2015)	rehabilitation services	initial days after the earthquake
Hurricane/ Typhoon	Katrina (2005)	Physical medicine and rehabilitation, physical therapy, occupational therapy and cast-application	First days
	Haiyan (2013)	Physical medicine and rehabilitation, physical therapy and occupational therapy	during the acute response up to five months
Emergency	Bombing	Physical therapy	Hospitalization
	Sri Lanka conflict	Spinal cord injury rehabilitation	After ending conflict

## Discussion

The main aim of the study was identifying characteristics of physical rehabilitation services in past disasters and emergencies. A number of included articles for review obviously showed that research related to disaster physical rehabilitation was limited and interest in research on disaster rehabilitation relief has been recently increased. Therefore, before 2012 research on this subject was sporadic.

The few publications on disaster physical rehabilitation are supported by some past studies (16, 17, 19–21). From among them, a few have presented the established characteristics of physical rehabilitation in disasters or emergencies. Almost all the articles included in this study have been written by authors who themselves played a part in providing the services after a disaster.

This study showed that physical rehabilitation reliefs were conducted sporadically in past disasters and emergencies. Emergencies, and that the services provided, especially during the early phase, were by and large, uncoordinated.

Our findings confirm that physical rehabilitation relief is either not provided or provided insufficiently during the early phases of disasters. After the Bam earthquake, the first rehabilitation specialists entered the earthquake zone after at least one month, and this delayed arrival was of little help to patients due to a lack of physical rehabilitation equipment (11, 22). In the Kashmir earthquake, rehabilitation services were provided for the patients in the first phase at the same time as medical treatment services. In spite of the timely response, however, there were rehabilitation related deficiencies and a lack of necessities in the region (19, 21). Moreover, because of poor conditions in Haiti, rehabilitation services were problematic and sporadic (18).

Among the different aspects of therapy, physical therapy and prosthetics were the most available needs for disaster and emergency victims (23, 24). Physical therapy is the most known in the fields of physical rehabilitation and prosthetics fabrication and is supported by international humanitarian organizations (7, 20). In the disasters of the past decade, rehabilitation aids have been established by national and international skilled or inexperienced organizations in the affected areas. This finding was stated in a pilot study conducted after the Haiti earthquake (25). Nearly all disaster physical rehabilitation services provided by national organizations or specialists in developing countries were not predicted before (14). Most times, rehabilitation specialists have provided services based on humanitarian reasons in disaster-affected areas. While optimum rehabilitation outcomes require a multidisciplinary approach, this is not possible without organizational responsibility (14, 26, 27). Hence, physical rehabilitation reliefs require regular and coordinated intervention to achieve optimal outcomes. Enough documentation does not exist on organizational efforts in developing disaster rehabilitation teams in national and international levels. Although, rehabilitation professionals have provided services to victims in more disasters in the past, the services have been rarely provided by non-specialist organizations or people (14). At present, rehabilitation resources are not able to meet rehabilitation needs, especially in the developing world.

Health system response to disasters requires disaster preparedness on three levels: prevention, medical treatment and rehabilitation (28). Many studies have been carried out on prevention, and medical treatment as well as developed models of service delivery for these two components (29–32). However, as also indicated, no such model is available in the literature regarding rehabilitation, especially in the early phases (23).

## Conclusion

Research on disaster physical rehabilitation is relatively new and limited evidence exist. Moreover, many of the existing studies in this scope are experience-based. This review recommends the integration of physical rehabilitation interventions into response plans of the health system at the time of disasters and emergencies. This issue needs further studies to be able to create a comprehensive model to deliver coordinated and appropriate services of physical rehabilitation following disasters and emergencies, so does the other healthcare models.

## Ethical considerations

Ethical issues (Including plagiarism, informed consent, misconduct, data fabrication and/or falsification, double publication and/or submission, redundancy, etc.) have been completely observed by the authors.
